# The Kinetics of Carrier Trap Parameters in Na_8_Al_6_Si_6_O_24_(Cl,S)_2_ Hackmanite

**DOI:** 10.3390/ma16206776

**Published:** 2023-10-20

**Authors:** Sung-Hwan Kim, Seon-Chil Kim

**Affiliations:** 1 Department of Radiological Science, Cheongju University, Cheongju 28503, Republic of Korea; kimsh@cju.ac.kr; 2 Department of Biomedical Engineering, Keimyung University, Daegu 42601, Republic of Korea; 3 Department of Medical Informatics, School of Medicine, Keimyung University, Daegu 42601, Republic of Korea

**Keywords:** hackmanite, tenebrescence, thermoluminescence, activation energy, F-center

## Abstract

Tenebrescence has been reported to have a high potential for personal ultraviolet (UV) detection. Color changes can detect UV doses and can also be used as visual sensors for X-rays. Hackmanite is known to exhibit tenebrescence. This study investigated the kinetics of electron-trapping levels contributing to the luminescence of Na_8_Al_6_Si_6_O_24_(Cl,S)_2_ hackmanite using thermoluminescence. The glow curves were measured at a heating rate of 5 K/s on hackmanite irradiated with X-rays. The physical parameters of the electron-trapping levels were evaluated by analyzing them using the deconvolution, peak shape, and initial rise methods. The Na_8_Al_6_Si_6_O_24_(Cl,S)_2_ hackmanite had at least five trapping levels, with activation energies of 0.78, 1.12, 1.86, 1.26, and 1.18 eV and corresponding peak trap lifetimes of 3.59, 2.71, 1.47, 3.34, and 3.91 s, respectively. The estimated migration time was 15.0 s.

## 1. Introduction

Hackmanite is primarily formed in low-silica and highly alkaline magma. It is commonly found within the cavities of volcanic blocks that have erupted from rock types, such as syenite and phonolite, and in metamorphosed limestone [1]. Hackmanite is also called tenebrescent sodalite or hackmanite, owing to its properties [2,3]. Among these, hackmanite has optical properties, such as photoluminescence and phosphorescence, and is known to exhibit reversible photochromism when exposed to ultraviolet (UV) and X-rays [4]. This phenomenon is called tenebrescence, which is based on the formation of color centers (F-centers) that trap electrons at defect energy levels with a band gap [2,5,6]. Tenebrescence has been commercially utilized in manufacturing lenses, clothing, filters, and smart coatings, and a high potential for personal UV detection has been reported [4]. Just as simple color changes can indicate UV doses, they can also be used as visual sensors for X-rays [7]. The X-ray-induced coloring of minerals was first reported in the 1950s, and since the late 20th century, it has been widely reported in various inorganic materials [4,8,9]. Recently, research on X-ray-induced photochromism for personal X-ray visualization has focused on metal–organic systems [10,11,12,13,14]. Tenebrescence occurs when a material is exposed to UV radiation, where chloride conductors capture the electrons of anionic polysulfide species (e.g., $S_{2}^{2-}$) [4]. Visible light and heat can re-emit electrons to the valence band, allowing the material to return to its original color; thus, these F-centers are quasi-stable. The lattice defects caused by electron or hole traps and the intentionally added impurity ions within the material cause continuous coloring. The yield of continuous luminescence owing to trapped electrons depend on the activation energy and frequency factor of the trap [13].

This study elucidates the physical characteristics of the electron trap levels that contribute to X-ray-induced coloring in Na_8_Al_6_Si_6_O_24_(Cl,S)_2_ hackmanite using thermoluminescence phenomena. The activation energy (*E*), frequency factor (*s*), and kinetic order (m) contributing to luminescence were determined using the peak shape, initial rise, and deconvolution methods.

## 2. Materials and Methods

### 2.1. Theory

**Figure 1:**
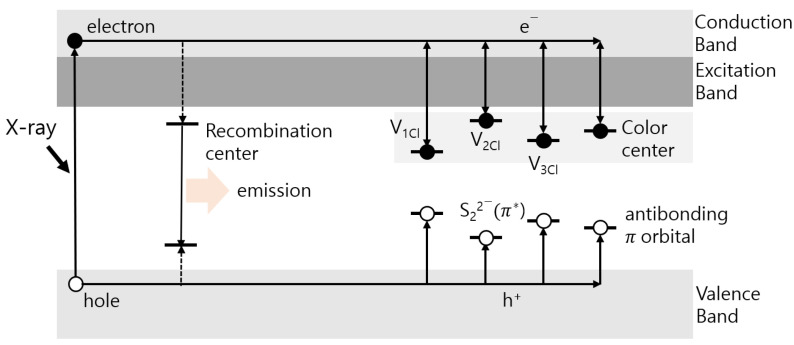
Schematic diagram of the energy band of the persistent luminescence processes.

As shown in Figure 1, the tenebrescence mechanism involves the trapping of electrons by Cl^−^ vacancies, which create color centers owing to the UV exposure of $S_{2}^{2-}$ ions that absorb energy in the visible light range [1]. When sufficient energy is provided, the captured electrons recombine at the luminescence center and return to the hydrosulfide. Na_8_Al_6_Si_6_O_24_(Cl,S)_2_ hackmanite also exhibits a characteristic orange/red luminescence when exposed to UV radiation, owing to the transition of the $S^{2-}$ lattice [1]. In this case, afterglow is observed for several seconds or longer. This phenomenon is called phosphorescence or permanent luminescence. Afterglow is caused by the continuous luminescence of charge carriers that are captured and released slowly rather than forbidden transitions (as in the case of phosphorescence) and always lasts longer than the emission lifetime of the emitting ion. Energy can be stored in a persistent luminescent phosphor, owing to the electron or hole traps within the material. Trap levels are related to lattice defects and intentionally added impurity ions, and exist as single or multiple traps. Electron carriers trapped at the trap levels can be activated through high-temperature thermal stimulation. They emit light through recombination from the trap levels, a phenomenon called thermoluminescence (TL). The yield of continuous luminescence within a phosphor strongly depends on the activation energy (depth of the trap) and frequency factor within the capture trap [13].

The physical parameters of the captured electron trap level can be determined by analyzing the TL phenomenon. TL is a phenomenon in which electrons from the valence band that absorb the energy of radiation irradiated to a medium are excited to the conduction band and then trapped at the electron trap level. When heat is applied, the trapped electrons are re-excited and emit light as they descend to the ground level [15,16]. Luminescence is used in personal dosimetry because the amount of luminescence generated at this time is proportional to the radiation dose. The temperature applied to the sample determines the amount of light emitted, expressed as a TL glow curve (glow curve) and can be used to obtain physical information regarding the electron trap level [17]. There are various methods for analyzing TL glow curves, such as the peak shape [18], initial rise [19], and heating rate [20] methods. Physical parameter analyses of trap levels using artificial intelligence libraries have also been performed [21]. This study used the peak shape and initial rise methods to determine the physical parameters of the trap level. The peak shape method proposed by Chen [20] determines the physical parameters of a TL electron trap level by obtaining the values of *ω*, *τ*, and *δ*, which are parameters of the peak at the center temperature (*T_g_*) of a single TL intensity curve and at points corresponding to half the height of the TL intensity. In general-order TL, when electrons trapped in the trap level emit light through thermostimulation, a phenomenon in which some of these electrons are recombined to the trap level also occurs. The values of TL peak parameters *ω*, *τ*, and *δ* are determined based on the recombination rate, and the higher the recombination rate, the higher the value of the symmetry factor (*μ_g_* (*=ω*/*δ*)). Chen [20] determined the activation energy (*E*) of the TL peak using three parameters of the TL peak, combining Equations (1)–(3) into a single TL glow curve peak, and obtained the kinetic order (m) and frequency factor (*s*) using Equations (4) and (5), respectively.

(1) $$E_{\omega }=\left[2.52+10.2\left(\mu_{g}-0.42\right)\right]\frac{kT_{g}^{2}}{\omega }-2{kT}_{g},$$

(2) $$E_{T}=\left[1.51+3\left(\mu_{g}-0.42\right)\right]\frac{kT_{g}^{2}}{\tau }-\left[1.58+4.2\left(\mu_{g}-0.42\right)\right]2{kT}_{g},$$

(3) $$E_{\delta }=\left[0.976+7.3\left(\mu_{g}-0.42\right)\right]\frac{kT_{g}^{2}}{\delta },$$

(4) $$m=e^{\left(-2.962+7.064\mu_{g}\right)},$$

(5) $$s=e^{\left(\frac{E}{{kT}_{g}}\right)}\frac{\beta }{kT_{g}^{2}}{\left(1+\frac{2{kT}_{g}}{E}\right)}^{-1},$$

where *E* is the activation energy (eV), *k* is the Boltzmann constant at 8.617 × 10^−5^ (eV/K), *μ_g_* is the symmetry factor (=*ω*/*δ*), *T* is the heating temperature, *T_g_* is the peak temperature of the glow peak (K), m is the kinetic order, *s* is the frequency factor (s^−1^), and *β* is the heating rate (K/s). The activation energy (*E*) refers to the depth of electron traps contributing to TL. The kinetic order (m) is a physical quantity representing the luminescence and recombination of electrons trapped in TL; it increases for a significant amount of recapture. Frequency factors indicate the frequency of electrons trapped at the capture level.

Rawat et al. [19] proposed the initial rise method for determining the activation energy of the trap level from the initial rise in the TL glow curve. The initial rise method can be used to determine the activation energy using Equation (6) in the early stages of the increasing intensity of the TL glow curve. From Equation (6), the activation energy can be determined from the slope of a straight line on a graph of the logarithm value ln[*I*(*T*)] of TL intensity against 1/*T*.

(6) $$\ln\left[I\left(T\right)\right]=-\frac{E}{kT}+C,$$

where *E* is the activation energy of the trap level (eV), *I*(*T*) is the TL intensity of the glow curve according to the heating temperature, *k* is the Boltzmann constant at 8.617 × 10^−5^ (eV/K), and *T* is the heating temperature (K).

The activation energy and frequency factor of the trap level determine the lifetime (*τ*) of electron carriers trapped at the trap level, as expressed in Equation (7) [22].

(7) $$\frac{1}{\tau_{trap}\left(T,E\right)}={s\left(r\right)e}^{-\frac{E}{kT}},\;\tau_{mig}=\sum_{i}\tau_{i},$$

where $\tau_{trap}$ is the trap lifetime (s), $\tau_{mig}$ is the migration time (s), *E* is the activation energy (eV), *T* is the temperature (K), *k* is the Boltzmann constant at 8.617 × 10^−5^ (eV/K), and *s* is the frequency factor (s^−1^).

### 2.2. X-ray Diffraction (XRD) of Na_8_Al_6_Si_6_O_24_(Cl,S)_2_ Hackmanite

**Figure 2:**
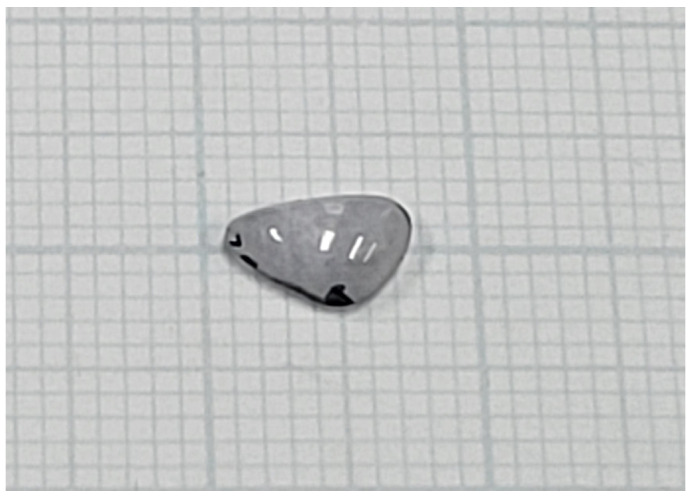
Photograph of the Na_8_Al_6_Si_6_O_24_(Cl,S)_2_ hackmanite sample.

**Figure 3:**
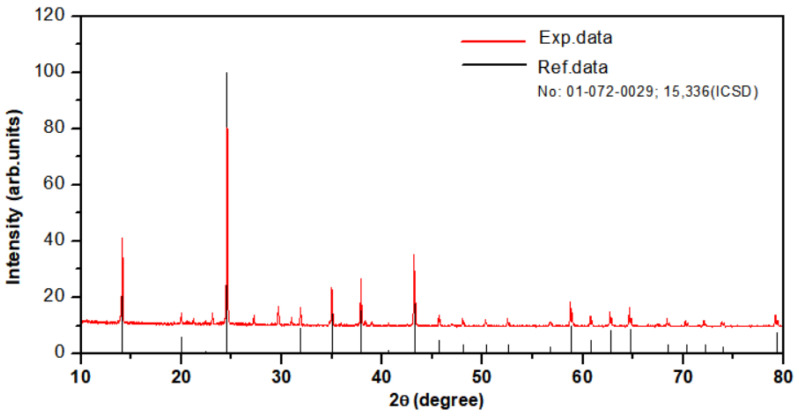
XRD powder diffraction patterns of the hackmanite.

Figure 2 shows a photograph of the hackmanite crystals used in this study. This crystal was pale gray, 7 mm long, 5 mm wide, and 3 mm thick. The structure and phase composition of the Na_8_Al_6_Si_6_O_24_(Cl,S)_2_ hackmanite sample were determined through powder XRD using a Shimadzu XRD-6100 X-ray diffractometer (Kyoto, Japan) with a Cu-K_α_ X-ray source. The XRD pattern was measured in the 2θ range of 10° to 80°. The XRD pattern of the sample, as shown in Figure 3, was well indexed to the same structure as that of reference No. 01-072-0029;15336 (ICSD), except for a few peaks at approximately 30°. The crystal structure and space group of the hackmanite are cubic, with a lattice constant of 8.870 Å and (218) [23,24].

### 2.3. TL Reader

**Figure 4:**
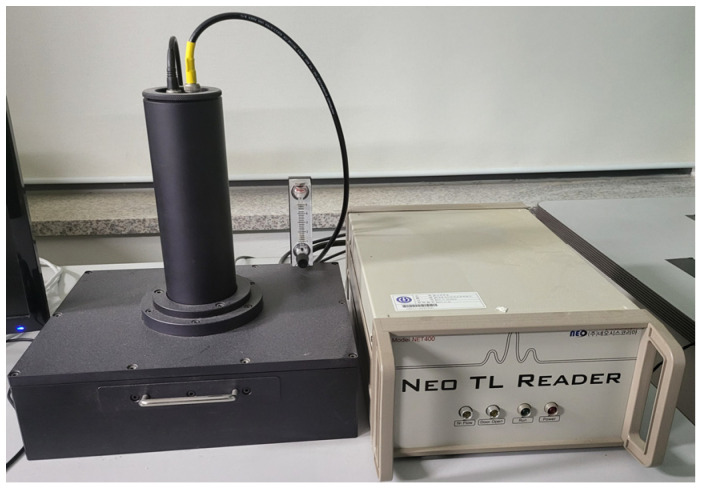
Neo TL reader (Neosis Korea Co., Daejeon, Republic of Korea).

Figure 4 shows the reader (Neosis Korea, Neo TL reader, Daegeon, Republic of Korea) used to obtain the TL measurements of the Na_8_Al_6_Si_6_O_24_(Cl,S)_2_ hackmanite sample at room temperature with a fixed heating rate of 5 K/s. X-rays were irradiated using a diagnostic X-ray generator (MIS Co., Daegeon, Republic of Korea) with a tube voltage, a tube current, and an irradiation time of 70 kV, 200 mA, and 0.2 ms, respectively. The exposure at a distance of 100 cm was 76 mR. The measured glow curve was separated through deconvolution using the TL/OSL Glow Curve Analyzer (ver. 1.1.0), and each peak was analyzed [25,26].

## 3. Results and Discussion

### 3.1. Measurement of the TL Glow Curve of Na_8_Al_6_Si_6_O_24_(Cl,S)_2_

**Figure 5:**
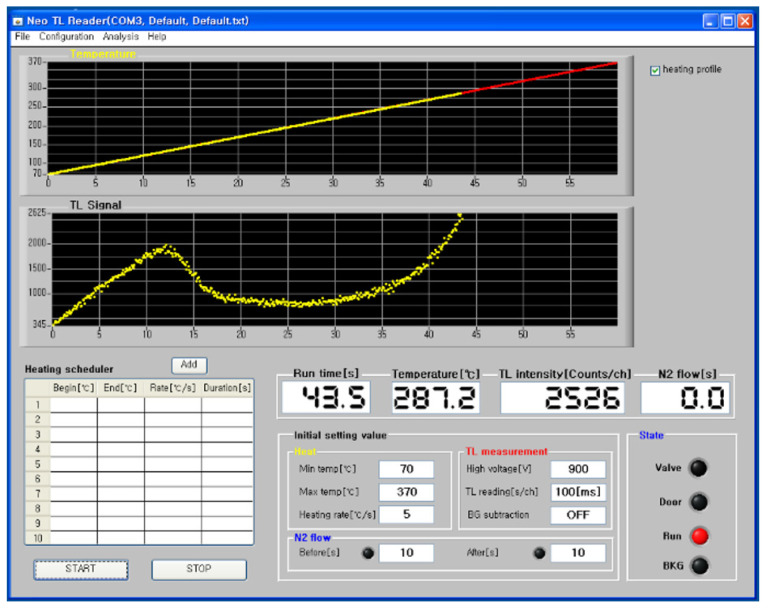
Glow curve of Na_8_Al_6_Si_6_O_24_(Cl,S)_2_ and graphical user interface of the TL measurement system (Neosis Korea Co. Daejeon, Republic of Korea).

Figure 5 shows the temperature over time and the TL glow curve of the Na_8_Al_6_Si_6_O_24_(Cl,S)_2_ hackmanite, measured using the Neo TL reader. The TL glow curve was measured from room temperature to a maximum of 570 K at a heating rate (*β*) of 5 K/s. The upper graph in Figure 5 presents the heating temperature measurement of the TL reader, and the sample temperature linearly increases in the measurement range from 273 K to 643 K. The lower graph in Figure 5 shows the glow curve measured at this time, and the TL measured data sampling rate was 10 Hz. The measured TL glow curve of the Na_8_Al_6_Si_6_O_24_(Cl,S)_2_ crystal comprised multiple peaks. The TL glow peaks were analyzed after removing the background.

### 3.2. Analyzed TL Trap Parameters through the De-Convolution Method

**Figure 6:**
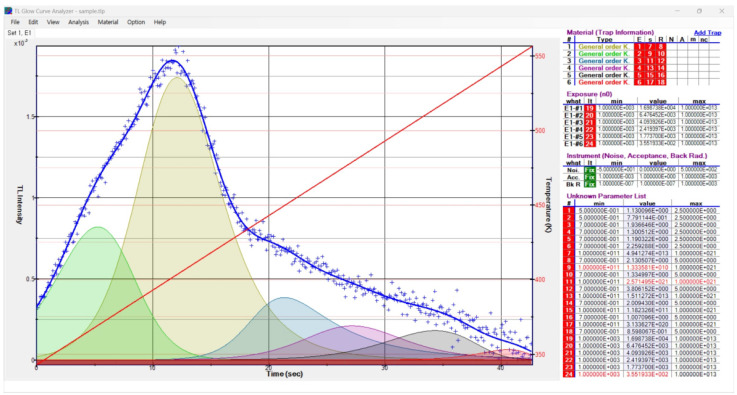
TL trap parameters of the glow peaks using the TL/OSL Glow Curve Analyzer software (version 1.1.0) [25,26].

Figure 6 shows the TL glow curve of the Na_8_Al_6_Si_6_O_24_(Cl,S)_2_ hackmanite after removing the background, which was separated into glow peaks using the TL/OSL Glow Curve Analyzer (ver. 1.1.0) software. As shown in Figure 6, five of the six separated peaks, accounting for 99% of the total luminescence, were determined as the main peaks contributing to hackmanite’s thermoluminescence, and, at this time, the figure of merit (FOM) representing the goodness of fit of the deconvolution data was 4.9% [27]. At least five peaks were observed, indicating the existence of over five trap levels with different energy levels. Table 1 lists the physical parameters of the five main traps determined through deconvolution. For the second peak, which contributed the most to luminescence, the activation energy of the trap level was approximately 1.13 eV and the frequency factor was 4.94 × 10^13^ s^−1^.

### 3.3. Analysis of TL Trap Parameters through the Peak Shape Method

**Figure 7:**
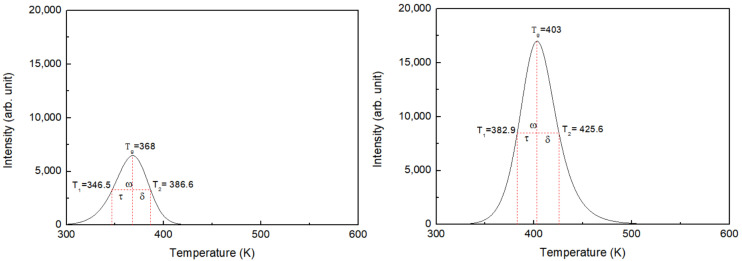
Glow curve and first and second peak parameters of the Na_8_Al_6_Si_6_O_24_(Cl,S)_2_ hackmanite obtained at a 5 K/s heating rate using the peak shape method.

Figure 7 shows the peak shape analysis results of peaks 1 and 2, that is, the main peaks of the Na_8_Al_6_Si_6_O_24_(Cl,S)_2_ hackmanite. Using a heating rate of 5 K/s, the peak temperatures obtained for peaks 1 and 2 were *T*_1*g*_ = 365 K and *T*_2*g*_ = 403 K, respectively, and the half-widths of the peaks were ω_1_ = 40.1 K and *ω*_2_ = 42.7 K, respectively. The symmetry factors for peaks 1 and 2 were *μ*_1*g*_(*δ*_1_/*ω*_1_) = 0.46 and *μ*_2*g*_(*δ*_2_/*ω*_2_) = 0.53, respectively. The activation energies, kinetic orders, and frequency factors of the Na_8_Al_6_Si_6_O_24_(Cl,S)_2_ hackmanite trap levels were determined through the peak shape method using Equations (1)–(5) and are listed in Table 2.

### 3.4. Analysis of TL Trap Parameters through the Initial Rise Method

**Figure 8:**
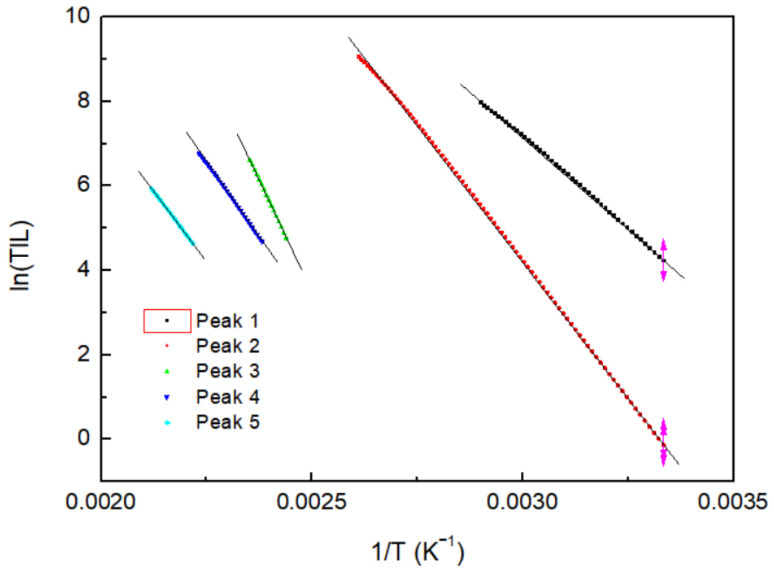
Activation energies of the Na_8_Al_6_Si_6_O_24_(Cl,S)_2_ hackmanite, determined through the initial rise method.

The initial rise method can determine the activation energy regardless of the kinetic order from the initial temperature rise during the heating of the TL glow curve. Figure 8 shows the fitting of ln (*I*(*T*)) vs. 1/T for the initial increase in the peaks in the TL glow curve of the Na_8_Al_6_Si_6_O_24_(Cl,S)_2_ hackmanite. The activation energy was determined from the slope of the fitted linear function. Table 2 lists the activation energies of the five peaks of the Na_8_Al_6_Si_6_O_24_(Cl,S)_2_ hackmanite that were determined through the initial rise method. The activation energies of 0.75, 1.11, 1.85, 1.21, and 1.14 eV are similar to the results obtained through the peak shape method.

Hackmanite is known to exhibit color-change characteristics when exposed to radiation and slow emission (afterglow), owing to a temporal delay. These characteristics are attributed to the activity of electrons captured at the trap level. The Na_8_Al_6_Si_6_O_24_(Cl,S)_2_ hackmanite TL trap level parameters obtained through the deconvolution, peak shape, and initial rise methods were within an acceptable error range. The initial rise method is generally known to measure slightly lower temperatures than other measurement methods because it analyzes the initial temperature rise during heating [19]. The experimentally determined lifetimes of the peak traps were 3.59, 2.71, 1.47, 3.34, and 3.91 s, and the migration time estimated using Equation (7) was 15.0 s.

## 4. Conclusions

This study measured a TL glow curve with a peak at 402 K at a 5 K/s heating rate to analyze the electron capture traps of Na_8_Al_6_Si_6_O_24_(Cl,S)_2_ hackmanite. The analysis using the deconvolution method revealed that the glow curve of Na_8_Al_6_Si_6_O_24_(Cl,S)_2_ hackmanite had over five trap levels contributing to luminescence. The physical parameters of each trap level were determined. The trap level depth yielded the same results within the acceptable error range for the deconvolution, peak shape, and initial rise methods. In addition, a direct correlation between the activation energy and frequency factor of the trap level and the lifetime of each trap was confirmed. A migration time of 15.0 s was determined, and a correlation between the trap level contributing to the persistent luminescence and the TL of Na_8_Al_6_Si_6_O_24_(Cl,S)_2_ hackmanite was confirmed.

## Figures and Tables

**Table 1:** Physical parameters of the Na_8_Al_6_Si_6_O_24_(Cl,S)_2_ hackmanite determined through deconvolution using the TL/OSL Glow Curve Analyzer (FOM = 4.9%).

Peak	Activation Energy (*E*) (eV)	Frequency Factor (*s*)(s^−1^)
1	0.78	1.33 × 10^10^
2	1.13	4.94 × 10^13^
3	1.94	2.57 × 10^21^
4	1.3	1.51 × 10^13^
5	1.19	1.18 × 10^11^

**Table 2:** Physical parameters of the Na_8_Al_6_Si_6_O_24_(Cl,S)_2_ hackmanite determined through the peak shape and initial rise methods. * FOM = 4.9%.

Peak	Trap Lifetime (s)	Temperature (K)						*μ_g_*	Activation Energy (eV)			
		T_1_	T_g_	T_2_	*ω*	*τ*	*δ*		Peak Shape	Initialize (R^2^ = 0.998)	Deconv. Method *	Avg.
1	3.59	346.5	368	386.6	40.1	21.5	18.6	0.46	0.80 ±0.02	0.75	0.78	0.78 ±0.02
2	2.71	382.9	403	425.6	42.7	20.1	22.6	0.53	1.12 ±0.02	1.11	1.13	1.12 ±0.02
3	1.47	435.4	453	479.8	44.4	17.6	26.8	0.60	1.78 ±0.05	1.85	1.94	1.86 ±0.06
4	3.34	453.6	478	504.3	50.7	24.4	26.3	0.52	1.28 ±0.01	1.21	1.3	1.26 ±0.04
5	3.91	488.3	514	533.1	44.8	25.7	19.1	0.43	1.22 ±0.01	1.14	1.19	1.18 ±0.03

## Data Availability

The datasets used and/or analyzed during the current study are available from the corresponding author on reasonable request.
